# The perceptions of first nation participants in a community oral health initiative

**DOI:** 10.1080/22423982.2017.1364960

**Published:** 2017-08-31

**Authors:** Kavita R. Mathu-Muju, James McLeod, Leeann Donnelly, Rosamund Harrison, Michael I. MacEntee

**Affiliations:** ^a^ Faculty of Dentistry, University of British Columbia, Vancouver, Canada; ^b^ First Nations Inuit Health Branch, Winnipeg, Canada

**Keywords:** Community health workers, dental primary prevention, pediatric dentistry, Aboriginal health, First Nations health

## Abstract

The Children’s Oral Health Initiative (COHI) is a federally funded community-based preventive dental program for children and their caregivers living in geographically isolated Canadian Aboriginal communities. The goal of the program is to improve access to preventive dental services for children of 0–7 years of age. It utilises community health workers in collaboration with dental therapists to promote and deliver the program. Almost half of the province of Manitoba’s (n=27) First Nations communities have implemented COHI since 2005. The objective of this investigation was to explore the opinions of COHI from the perspective of community members whose children had participated in the program. Purposeful selection identified caregivers of enrolled children for a semi-structured interview. The targeted caregivers had children who met at least one of the following criteria: (1) 0–2 years old; (2) 5–7 years old; (3) had two or more children either currently or formerly enrolled in COHI. Six open-ended questions guided the interview process. Content analysis was used to code transcripts and identify themes. One hundred and forty-one interviews were completed in 13 communities. Participants defined good oral health as the absence of dental cavities, which reflects a Western biomedical model of disease. The local, community-based nature of COHI was viewed as essential to its success in increasing access to preventive dental services and improving children and caregivers’ oral health knowledge and behaviours. In conclusion, a local, community-based oral health prevention programme is perceived as having a beneficial effect on children and caregivers’ oral health knowledge and behaviours. However, oral health preventive messages need to be further integrated into traditional Aboriginal holistic models of wellness.

## Background

The Children’s Oral Health Initiative (COHI) was initiated and funded by Health Canada in 2004 as a community-based preventive dental health programme for children and their caregivers living in remote communities in Canada. The goal of the programme is to improve access to preventive dental services for children [1]. The initiative is offered to communities through a participatory process of consent from each community, whereby, in accordance with Articles 19 and 23 of the UN Declaration on the Rights of Indigenous Peoples 2007, the communities assume control over decisions influencing the implementation and operations of the programme, including the decision to terminate it [1,2]. This initiative attempts to harmonise the indigenous people’s traditional healthcare practices with western dental medicine, to enhance the oral health of Aboriginal children through a co-management approach to delivering care in each community [3].

Almost half of all 636 First Nations and Inuit (FN/I) communities across Canada have opted to implement the initiative since it was piloted in 2004 [1]. It is delivered by COHI aides, who are lay community oral health workers living in the communities. They collaborate with externally contracted dental therapists and dental hygienists to screen 0–7 year old children for dental caries, provide preventive oral health information, apply fluoride varnish and refer children to the dental therapist or hygienist for sealants and atraumatic restorative treatment (ART) or to a dentist for further complex treatment, as needed [4]. Little is known about the perspectives of Aboriginal families who enrolled their children in the COHI programme. Understanding local processes of knowledge dissemination and utilisation may provide insight into whether the programme has translated effectively into communities. Therefore, the objective of this investigation was to explore the experiences and opinions of First Nations families whose children had enrolled in the Children’s Oral Health Initiative.

## Methods

### Development of the research process

The investigation was initiated by COHI staff and community members from Manitoba Region, First Nations Inuit Health Branch. Although nearly half (n=27) of the province’s 63 First Nations (FN) communities had consented to continuously implement COHI since 2005, no formal assessment had been conducted to obtain community feedback on the programme. Consequently, the Manitoba Dental Programme Manager contacted previous collaborators at the University of British Columbia (UBC) for assistance in helping design and implement an evaluation strategy. A 16 member advisory group was formed that included: the Dental Programme Manager, two FN community-based dental therapists, 10 individuals who worked with FN communities in health planning or delivery and three university-based public health researchers. A workshop was held to develop an approach to explore the experiences and opinions of participants in COHI and to seek their advice on improving the programme. The approach agreed upon was a co-operative inquiry that engaged community members in a collaborative interview process [5]. The advisory group developed six open-ended questions to guide the interviews (Table 1) [6]. The group also identified suitable communities, recommended recruitment methods and provided advice on how to record the participants’ responses.

**Table 1.** Interview questions.

Question 1	Tell me how you would describe a healthy mouth?
Question 2	Could you tell me three things you learned from the COHI programme?
Question 3	Do you think your child has better oral health from participating in this programme?
Question 4	Can you tell me how this programme works for you?
Question 5	How does this programme benefit the community?
Question 6	Is there anything about the programme we could improve?

### Sampling and recruitment

The advisory group recommended purposeful selection to identify parents, grandparents and others who served as caregivers for enrolled children for the semi-structured interviews [6]. The targeted caregivers had children who met at least one of the following criteria: (1) 0–2 years old; (2) 5–7 years old; or (3) had two or more children either currently or formerly enrolled in COHI. The purpose of the sampling strategy was to interview caregivers of children from all age groups eligible for the programme and to provide at least two participants from each category of caregiver. This approach allowed exploration of experiences from caregivers whose children received predominantly preventive care at home (0–2 year olds) vs. at school (5–7 year olds), as well as caregivers whose children had “aged out” of the programme. Thirteen rural communities geographically isolated from year-round health services were selected: four were accessible only by air travel year-round; five had year-round road access and required at least 2.5 hours of driving time to Winnipeg (urban centre with health services); and four had year-round road access and required at least 4 hours of driving time to Winnipeg.

The advisory group recommended that community-based COHI staff were in the best position both to recruit and interview participants because of their established relationships with the communities. The First Nations members of the advisory group strongly believed that interviewers who were unknown by the communities would have difficulty gaining the trust and support of community members. The investigation received the approval of the UBC Research Ethics Board.

### Interviews

The COHI staff in each of the selected communities attended a workshop on qualitative interviewing techniques. They received practical instruction on posing the six semi-structured interview questions. The First Nations community members in the advisory group had indicated that participants would not respond openly if the interviews were recorded on audiotape. Consequently, the interviewers were instructed on how to record the participants’ responses in detailed notes during the interview and how to document the main points of the interview immediately after it ended [6]. They were advised to probe for additional information in an open-ended and free manner by encouraging participants to speak freely with minimal interruption [7]. Participants were asked the same questions in the same sequence to facilitate analysis [6]. The interviews occurred in a quiet and private space within homes, schools, health centres or nursing stations at the convenience of each participant.

### Data management and content analysis

The interviewers’ notes were transcribed to a Word document. Two researchers independently read the transcripts and, through a process of content analysis, coded each transcript to identify themes and summarised each participant’s perspectives. The emerging themes were refined further by consensus agreement between the pair of researchers, and representative samples of text were selected to illustrate the essence of each theme.

## Results

One hundred and forty-one interviews were completed in 13 communities. The majority (79%, n=111) of those interviewed were parents, followed by other relatives/caregivers (13%, n=18) and grandparents (9%, n=12). Thirty-five percent of participants (n=49) had two or more children either currently or formerly enrolled in COHI, 35% (n=50) had children who were 0–2 years old and 30% (n=42) had children who were 5–7 years old. The responses to Question 1: “Tell me how you would describe a healthy mouth?” tended to be short and descriptive. The most common response was “no cavities”, followed by other descriptors such as “clean”, “white teeth”, “healthy gums”, “no odour” and “no missing teeth”. One participant explained that a healthy mouth “is one that is brushed at least twice a day and flossed at least once a day”.

The educational role of COHI was addressed in Question 2: “Could you tell me three things you learned from the COHI programme?” Responses centred on preventing the development of oral diseases in infants. Participants described learning to clean infant teeth and gums “before teeth are even in the mouth” and to avoid potentially cariogenic feeding practices such as putting children to sleep with a bottle. As two participants explained:

It is a very good programme. It catches the small children at a young age for prevention and helps mothers and fathers like me who stay home to care for my little ones; to help us understand why we need to brush every day, the importance of clean and healthy mouth in babies – to prevent dental surgery in them.

I didn’t know before that you could place temporary fillings and plastic protection on kids’ teeth. I learned about a soft toothbrush of rubber to be used for babies just getting their teeth. I learned that juice in bottles is really bad for teeth and certain junk foods are bad and teeth must be brushed several times a day plus flossing, even for kids.

Others said that they learned how cups should replace bottles as soon as possible, and that “the bottle which has juice in it can cause damage to my baby’s teeth if they sleep with it”. They learned also that infants benefit from early professional examinations of their teeth and gums as well as topical applications of fluoride varnish.

The third question explored parents’ perception of COHI’s impact on dental disease in their children: “Do you think your child has better oral health from participating in this programme?” Answers were overwhelmingly positive. Parents and caregivers responded that the programme had improved their children’s oral health behaviours, especially tooth-brushing habits:

Yes, they are more aware of looking after their teeth because of the lessons learned from a professional person as opposed to parents nagging.

My child has benefitted from participating in this programme very much so that she has been brushing her teeth without being told.

Programme participation resulted in improved oral health knowledge for both children and parents, as demonstrated by comments such as: “my child is learning about things he can’t eat, that will ruin his teeth, like pop and chips” and “I am knowledgeable about looking for decay. I know the difference between teeth that have been brushed for days and that are brushed regularly. I ask my children, ‘do they feel like fuzzy slippers?’”

Finally, it was felt that the local, community-based nature of the programme improved access to preventive and restorative dental services, resulting in fewer cavities and less untreated dental disease in children:

Yes – my son got the cleaning and filling done here on reserve and that saved the problem from getting worse. It was recognised early and treated by the therapist before it became a bigger problem.

The child that was enrolled from birth has better oral health than older siblings, due to the oral health programme.

Question 4, “Can you tell me how this programme works for you?” elicited opinions regarding the effectiveness of the programme at the level of the individual child. The local, community-based nature of the programme was again identified by the majority of participants as the reason the programme was successful in providing preventive services for children. The need for travel to access regular preventive dental care was eliminated. Instead, an opportunity to develop an on-going therapeutic relationship with the dental therapist and local COHI Aide to promote continuity of care was provided. These relationships also facilitated referrals and linkage to the oral healthcare delivery system outside the community when needed: “I was able to get a quick referral from the dental clinic/dental therapist. If I went to The Pas, there is a long wait list for a GA [general anaesthesia] referral”.

Preventive behaviours at home were reinforced because the local programme staff supported caregivers by “educating me about what can I do to help my children to look after their teeth“, as well as providing dental supplies to implement tooth-brushing at home: “Receiving a tooth brush and tooth paste for my child’s teeth encourages them to brush more often”.

The COHI was valued for helping eliminate dental pain and disease “I don’t like my children to have pain or cavities. It works for me so they feel better” and reduced the need for children to travel outside the community for full mouth dental rehabilitation under general anaesthesia: “There are not as many children going out for GA [general anaesthesia] surgery. There is a noticeable difference with children that take part in the COHI programme, vs. the ones that don’t take part”.

Question 5 asked “How does the programme benefit the community?” to acknowledge that Aboriginal people value collective approaches to health, because benefits to individuals are perceived to be integrated with gains for the entire community. The most predominant answer was that the programme increases access to preventive services because it takes place in the community and, therefore, provides continuity of care with a locally-based COHI Aide and dental therapist:

The services are here from pre-school. The children start at an early age. The children have less fear while attending their dental appointment. They learn not to fear the Dental Therapist/COHI Aide. Having built trust in the dental team helps the child be more willing to get their treatment done. The services are convenient and the office is open when we need them. Emergency situations are dealt with right away.

The programme was described as increasing both awareness and knowledge about oral health at the community level: “It helps us understand how important our oral health is; and that we should start keeping our mouths clean and healthy”. COHI helped decrease barriers to promote and maintain good oral health because “dental supplies are available and distributed to everybody who needs them”. Prioritising the well-being of the community’s children was considered a tangible benefit, resulting in improved oral health knowledge and behaviours: “It’s teaching the kids how to take care of their teeth to benefit their overall health”. It was noted that another benefit was reducing the number of children who needed to be sent out from the community for dental treatment with the use of general anaesthesia: “There are less parents taking their children to get GA surgery”. Parents believed that this resulted from implementing early preventive measures for 0–2 year olds, thus preventing the development of severe early childhood caries, as well as managing the cavitated lesions of pre-school/kindergarten aged children with minimally invasive dentistry (remineralisation with fluoride varnish or ART): “See lots of kids with lots of cavities; better to do fluoride and temporaries before major work [general anaesthesia]”.

The sixth and final question, *“*Is there anything about the programme we could improve?” sought community members feedback on COHI. Suggestions for improvement centred on increasing community level involvement; for example, hiring more local staff to increase the availability of services: “Hire another therapist/aide to provide COHI treatment only. This community is large. We could use more therapists”. It was also felt that local staff could be better utilised to improve community awareness of the programme and its objectives: “Maybe the COHI lady can do more public things. Like go on the radio station or TV station”. Another suggestion to raise the programme’s profile was to combine COHI with other community-based programmes to increase the programme’s scope: “maybe combine efforts with another programme to help increase awareness; for example, a dental visit with a well-baby clinic”. Finally, it was felt that consideration should be given to expanding the programme’s reach to include other vulnerable members of the community, specifically elders and pre-teenaged children.

## Discussion

The interviews provided caregivers an opportunity to express their opinions and experiences with COHI in their own communities. This engagement approach was designed and undertaken at the request and input of those directly involved with the programme. It allowed exploration of community members’ perspectives as to how COHI has influenced their oral health knowledge and behaviours and whether they considered programme participation beneficial for their children.

A significant finding from the interviews was that a “healthy mouth” was defined as one that had “no cavities”, “white teeth” and “healthy gums”; in other words, one that was free of disease. This has implications for programme planning. A challenge faced by federal health policy planners has been past failure to acknowledge that Aboriginal culture defines health as a holistic balance of mental, physical, spiritual and emotional well-being that goes beyond the dominant Western biomedical model that sees health as simply “free from disease” [8]. As First Nations and Inuit (FN/I) people continue transitioning to self-governance in health, emphasis has been placed on the importance of traditional wellness as a means of improving health and quality-of-life for Aboriginal people [3]. Yet, the findings of this study demonstrated that participants did not identify oral health with a holistic model of wellness reflecting their traditional cultural values. This apparently contradictory outcome merits further exploration and explanation.

Insight into this finding is provided by an understanding of the aetiology of dental caries and the historical context of the disease in First Nations people. Dental caries, post-colonialism, is a chronic disease resulting from “modernization”, because it accompanies the lifestyle of Western industrialised nations [9]. The fossil record suggests that dental health during the pre-contact period was quite good, as dental caries affects less than 1% of all teeth in pre-colonial mummies and skulls from the North West Coast and the Artic [10]. The rise in tooth decay accompanied the development of other previously low prevalence diseases such as cardiovascular disease and diabetes, that were directly caused by drastic changes in lifestyle and diet [11,12]. Diets changed when treaty agreements substituted traditional hunting and gathering lifestyles with poor quality government food [12]. Access to healthy foods is a problem that continues to this day, especially in the rural reserves targeted by COHI. In other words, the high prevalence and severity of dental caries in FN/I populations today is related directly to food insecurity [13]. Ancestral land resources have been redirected to cash crops or other industrial uses, and are no longer available for their traditional use as a source of family food [11]. Therefore, it is not surprising that traditional models of wellness do not extend to describe a disease that was essentially non-existent prior to European colonialism. Hence, a default “Western” definition of a healthy mouth has emerged.

Caregivers described learning how to prevent the development of oral diseases in infants by, for example, changing cariogenic feeding practices such as putting a baby to sleep with a bottle. They also discussed learning the benefits of early professional dental examinations, as well as topical application of fluoride varnish and dental sealants. These results represent acquisition of knowledge about mainstream therapeutic and preventive oral health measures. This is a positive finding because it empowers the population to know and understand what services are available from COHI that could potentially benefit the oral health of the community. Utilisation and access to healthcare services are important determinants of health for both Aboriginal and non-Aboriginal Canadians [14]. These results support other reports which suggest traditional community-based healing and counselling is an effective way to manage health promotion and disease prevention by integrating new knowledge about dental caries that traditional healing methods have been unable to address [12,14].

Caregivers believed that participating in COHI had a beneficial effect on their children’s oral health, because it improved their understanding of managing dental disease relating to diet and tooth brushing behaviours. Children learned to identify cariogenic foods, a significant improvement since children with healthy diets are less likely to develop severe dental caries compared to children with poor dietary practices [15,16]. Parents attributed their children’s improved oral hygiene habits to the efforts of the local dental therapist and COHI Aide. This finding supports earlier research suggesting that individual counselling with regular follow-up by a lay person, such as the COHI Aide, is an effective way to facilitate adoption of constructive oral health behaviours [17]. Parents identified the local, community-based nature of the programme as being critical to this process; it allowed for children to have early access to preventive care, which in turn influenced programme participation and acceptance of COHI’s primary preventive educational messages [18].

The absence of continuity of delivery of health services has resulted in poor health outcomes in northern Aboriginal communities [19]. This explains participants repeatedly citing the local, community-based nature of the programme as the reason COHI was beneficial for both individual families and the collective community. The continuity of care provided by COHI staff improved early access to preventive services, reduced the need for travel to access oral healthcare and was also perceived as decreasing the need to send children outside the community for dental treatment under general anaesthesia. However, it was noted that the programme facilitated referrals and linkages to dental care outside the community when children required more complex dental treatment.

Suggestions on improving COHI focused on expanding it to include older children and elders and enhancing communications to promote greater awareness of the programme. A frequent recommendation was that the programme continue to provide toothbrushes and toothpaste at no cost to families, in order to enable implementing healthy brushing habits at home. Northern communities have an insufficient retail infrastructure, resulting in extraordinarily high costs for basic necessities such as groceries, baby products and personal hygiene products [20]. Provision of dental supplies was, therefore, an essential component of enabling children to implement the oral hygiene behaviours encouraged by the programme. Sustainability was enhanced once the barrier of exorbitantly priced basic goods was removed.

The main limitation of the study was potential bias from utilising local programme staff to conduct the interviews, which increased the likelihood of socially desirable responses [21]. However, the First Nations members of the advisory group felt strongly that unfamiliar interviewers would have difficulty gaining the trust and support of the communities to recruit participants for the project. Furthermore, it has been recognised that practitioner involvement in evaluation inquiries that are small scale, local and carried out by those directly involved in service delivery is essential to good professional practice [22]. The structure of the questions remained unchanged, because of geographical barriers inhibiting access to the interviewers’ notes until all interviews were completed. However, at least two caregivers from each interview group were identified during recruitment to help offset the inability to perform constant comparison during the interview process. Upon the recommendation of the community members in the advisory group, the interviews were recorded by hand. Taking extensive notes during an interview may be distracting and interrupt the free flow of conversation. Despite these limitations, consistent themes emerged across communities. Finally, as purposeful sampling was used to select communities, it cannot be assumed that the results are generalisable to all FN/I communities in other provinces and territories. The Children’s Oral Health Initiative has been implemented nationally in nearly half of Canada’s First Nations communities, and this study provides the first formal documentation of community members’ opinions of the programme.

## Conclusions

The results demonstrate that a local, community-based oral health prevention programme, utilising lay community health workers collaborating with dental therapists/hygienists, is welcomed and perceived as beneficial by First Nations community members. However, a western definition of oral health as “free from disease” predominates in communities. This suggests that oral health preventive messages need to be further integrated into traditional holistic models of wellness and should target the underlying reasons for dental caries, especially access to a healthy diet and issues of food insecurity. Although primary oral healthcare can be effective in mitigating the effects of poor diet and oral health behaviours, the high prevalence and severity of dental disease in FN/I children will continue until the underlying social determinants of health are effectively addressed. Traditional, community-based care is especially important in rural areas, where access to primary healthcare facilities is difficult and sometimes impossible. Programmes such as the Children’s Oral Health Initiative should be sustained and expanded.
